# 4099 Principles of Statistical Education for Translational Scientists in the Age of Rigor, Reproducibility, and Reporting

**DOI:** 10.1017/cts.2020.183

**Published:** 2020-07-29

**Authors:** Emilia Bagiella, Paul Christos, Mimi Kim, Shing Lee, Roger Vaughan, Judy Zhong

**Affiliations:** 1 Mount Sinai School of Medicine; 2 Albert Einstein College of Medicine; 3 Rockefeller University; 4 New York University General Clinical Research Center

## Abstract

OBJECTIVES/GOALS: To describe principles, best practices, and techniques recommended to instill deep understanding of the application and interpretation of statistical techniques and statistical inference among translational scientists and trainees, that best support the concepts of scientific Rigor, Reproducibility and Reporting. METHODS/STUDY POPULATION: Each of the six New York City Area Biostatistics, Epidemiology and Research Design (BERD) resources have strong educational programs, novel curricular components, and creative strategies, implemented by award winning educators. To capitalize on shared knowledge, innovation, and resources, the six teams formed the **New York City Area BERD Collaborative (NYC-ABC)** comprised of BERD resources from Mt. Sinai, Cornell, Einstein, Columbia, Rockefeller, and NYU. The collaborative suggests principles, concepts, tools and approaches to support the concepts of scientific Rigor, Reproducibility and Reporting in translational science. RESULTS/ANTICIPATED RESULTS: Principles:

- Value of team science approach and including biostatisticians early and often.
- Carefully designing experiments to reduce bias and increase precision.
- Trainees’ focus is often on “statistical significance” and the p-value. Consequences of data dredging/p-hacking, and the impact of sample size and other factors on statistical significance.
- Emphasizing the effect size and answering the scientific hypothesis when reporting results.
- Statistical code used to produce results should be well annotated and raw data posted online to enhance reproducibility.

Approaches:

- Incorporate effective multiple modalities (i.e. didactic, demonstrative, hands on workshops, applications, and tools).
- Approach from “the drivers’ seat” perspective, rather than strictly mathematical.
- Endorse flipped classroom approach

DISCUSSION/SIGNIFICANCE OF IMPACT: Like any complex discipline, biostatistical education can be approached from several dimensions, but it remains essential to focus on fundamental goals of science. We remind our trainees that the goal of science is to create knowledge, not to “find significance”. Deep understanding of inferential methods and proper interpretation of results are key. CONFLICT OF INTEREST DESCRIPTION: None.

